# Improving Community Health While Satisfying a Critical Community Need: A Case Study for Nonprofit Hospitals

**DOI:** 10.5888/pcd12.150230

**Published:** 2015-10-29

**Authors:** Alicia M. Hoke, Donna K. Kephart, Judith F. Dillon, Jody R. McCullough, Barbara J. Blatt, Jennifer L. Kraschnewski

**Affiliations:** Author Affiliations: Donna K. Kephart, Penn State Hershey PRO Wellness Center, Penn State College of Medicine, Hershey, Pennsylvania; Judith F. Dillon, Penn State Hershey Medical Center, Hershey, Pennsylvania; Jody R. McCullough, Barbara J. Blatt, Jennifer L. Kraschnewski, Penn State College of Medicine, Hershey, Pennsylvania.

## Abstract

**Background:**

School-based student health screenings identify issues that may affect physical and intellectual development and are an important way to maintain student health. Nonprofit hospitals can provide a unique resource to school districts by assisting in the timely completion of school-based screenings and meet requirements of the Affordable Care Act. This case study describes the collaboration between an academic medical center and a local school district to conduct school-based health screenings.

**Community Context:**

Penn State Milton S. Hershey Medical Center and Penn State Hershey PRO Wellness Center collaborated with Lebanon School District to facilitate student health screenings, a need identified in part by a community health needs assessment.

**Methods:**

From June 2012 through February 2013, district-wide student health screenings were planned and implemented by teams of hospital nursing leadership, school district leadership, and school nurses. In fall 2013, students were screened through standardized procedures for height, weight, scoliosis, vision, and hearing.

**Outcomes:**

In 2 days, 3,105 students (67% of all students in the district) were screened. Letters explaining screening results were mailed to parents of all students screened. Debriefing meetings and follow-up surveys for the participating nurses provided feedback for future screenings.

**Interpretation:**

The 2-day collaborative screening event decreased the amount of time spent by school nurses in screening students throughout the year and allowed them more time in their role as school wellness champion. Additionally, parents found out early in the school year whether their child needed physician follow-up. Partnerships between school districts and hospitals to conduct student health screenings are a practical option for increasing outreach while satisfying community needs.

## MEDSCAPE CME

Medscape, LLC is pleased to provide online continuing medical education (CME) for this journal article, allowing clinicians the opportunity to earn CME credit.

This activity has been planned and implemented in accordance with the Essential Areas and policies of the Accreditation Council for Continuing Medical Education through the joint sponsorship of Medscape, LLC and *Preventing Chronic Disease*. Medscape, LLC is accredited by the ACCME to provide continuing medical education for physicians.

Medscape, LLC designates this Journal-based CME activity for a maximum of 1 ***AMA PRA Category 1 Credit(s)™***. Physicians should claim only the credit commensurate with the extent of their participation in the activity.

All other clinicians completing this activity will be issued a certificate of participation. To participate in this journal CME activity: (1) review the learning objectives and author disclosures; (2) study the education content; (3) take the post-test with a 75% minimum passing score and complete the evaluation at www.medscape.org/journal/pcd; (4) view/print certificate.

**Release date: October 29, 2015; Expiration date: October 29, 2016**

### Learning Objectives

Upon completion of this activity, participants will be able to:

1. Assess health screening requirements encountered among schools in the United States.
2. Evaluate the practice of school health screening in the current study.
3. Distinguish the proportion of students reached by health screening in the current study.
4. Describe potential improvements to the screening protocol used in the current study.

**EDITOR**

Ellen Taratus, Editor, *Preventing Chronic Disease*. Disclosure: Ellen Taratus has disclosed no relevant financial relationships.

**CME AUTHOR**

Charles P. Vega, MD, Clinical Professor of Family Medicine, University of California, Irvine

Disclosure: Charles P. Vega, MD, has disclosed the following relevant financial relationships: Served as an advisor or consultant for: Lundbeck, Inc; McNeil Pharmaceuticals; Takeda Pharmaceuticals North America, Inc.

**AUTHORS**

Alicia M. Hoke, MPH, CHES; Donna K. Kephart, MHA; Department of Pediatrics, Penn State Hershey PRO Wellness Center; Penn State Hershey College of Medicine, Hershey, Pennsylvania.

Judith F. Dillon, MSN, MA, RN; Jody R. McCullough, AA; Barbara J. Blatt, M.Ed; and Jennifer L. Kraschnewski, MD, MPH, Penn State Hershey Medical Center; Nursing, Hershey, Pennsylvania.

Disclosure: Alicia M. Hoke, Donna K. Kephart, Judith F. Dillon, Jody R. McCullough, Barbara J. Blatt and Jennifer L. Kraschnewski have disclosed no relevant financial relationships.

## Background

The concept that healthier students are better learners is supported by leading national education organizations and a wide body of literature (1,2). Given that 91% of all US adolescents attend school, schools are an ideal setting for delivering services and programs to keep students healthy and ready to learn (3). One important intervention is school-based student health screenings, used by schools to identify potential medical concerns that might affect physical and intellectual development. Most US school districts have policies that require routine screening for vision impairment (90.3% of school districts) and hearing impairment (91.7% of school districts) (4). As a potential approach to addressing the childhood obesity epidemic, 18% of states have also mandated body mass index (BMI) screening in schools (4,5). School-based screening requires a significant effort by school personnel, often the school nurse. In some schools, an entire school year is needed to complete screenings and disseminate results to parents, thereby delaying any action that could correct potential problems.

Regulations under the Affordable Care Act require nonprofit hospitals to complete community health needs assessments and identify ways to satisfy unmet needs (6). School districts identified through a needs assessment as having unmet needs provide an opportunity for nonprofit hospitals to help.

This case study describes a novel collaboration between a medical center and a neighboring school district, in which hospital nursing leaders assisted in conducting school-based screenings. We propose that nonprofit hospitals are a unique resource to school districts for assistance in conducting school-based screenings (Figure).

**Figure:**
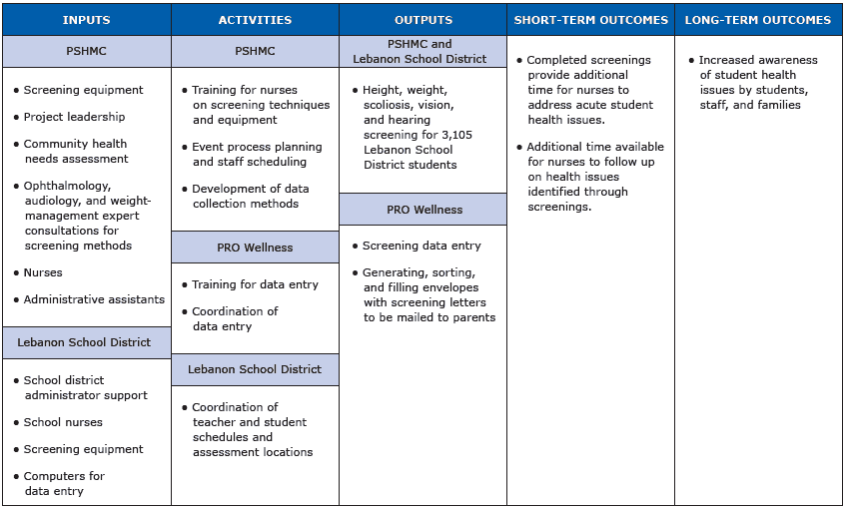
Logic model describing engagement of involved organizations and implementation of the Lebanon student health screenings, Lebanon, Pennsylvania, 2012–2013. Abbreviations: PSHMC, Penn State Milton S. Hershey Medical Center; PRO Wellness, Penn State Hershey PRO Wellness Center. [A text version of this figure is also available.]

## Community Context

A community health needs assessment was conducted in 2011 by a collaborative of hospital systems in Central Pennsylvania. The assessment included Cumberland, Dauphin, Lebanon, Perry, and York counties, all served by the health care systems involved. Assessment data showed that Lebanon county had greater health vulnerabilities than other counties assessed, including higher rates of children living in poverty and single-parent households, obesity, smoking, teen births, and uninsured families and lower proportions of primary care physicians and dentists (7). The needs assessment data correlated with the health rankings of Pennsylvania counties (8), demonstrating the effectiveness of the needs assessment in identifying the needs of the community. 

In 2012, Penn State Milton S. Hershey Medical Center (PSHMC) reached out to Lebanon School District, one of the main school districts in Lebanon County, to organize a collaborative effort to respond to the vulnerabilities identified. In this district, 74% of students were enrolled in the national program for free or reduced-price lunch, and 39% of students in kindergarten through grade 6 and 38% of students in grade 7 through grade 12 were overweight or obese (9,10). District nurses and administrators indicated that the length of time required to complete the annual student health screenings was taking away from time needed to manage the acute health needs of students. In addition, the screening process resulted in delays in the dissemination of critical health information to parents and care providers, thereby prolonging or reducing any response to the screening results.

Penn State Hershey PRO Wellness Center (PRO Wellness), an organization committed to educating and inspiring youth and their families to eat well, engage in regular physical activity, and become champions for bringing healthy choices to life, works primarily with schools in Pennsylvania to provide proven interventions and technical guidance in program planning, data collection, and reporting. A natural partnership emerged between PRO Wellness and the PSHMC community benefits team to facilitate student health screenings in Lebanon School District in support of PSHMC’s commitment to community activities.

## Methods

Three nonprofit hospitals (PSHMC, PinnacleHealth System, and Holy Spirit Health System) initiated the joint venture to conduct a community health needs assessment of the 5 counties they served. The assessment consisted of 1) interviews of personnel at public and private organizations, such as educational institutions, human services organizations, and faith-based organizations; health professionals; and local government officials; 2) focus groups with key audiences; and 3) community forums (eg, town hall–style meeting). The assessment also used data on disease prevalence, socioeconomic factors, and behavioral habits (7). The assessment identified 3 priority areas: promotion of healthy lifestyles, health education, and access to affordable health care. Lebanon County’s health needs were related to health insurance, poor access to health care, overweight and obesity, and health education (7).

The screening initiative was built on an established relationship between PSHMC and PRO Wellness and the Lebanon community. The school district is home to nearly 5,000 students and comprises 5 elementary schools, 1 middle school, and 1 high school. School administrators and nurses identified a hardship in completing the annual student health screenings required by the Pennsylvania Public School Code (11). Nurses from PSHMC and PRO Wellness staff collaborated on a plan to conduct screening events that met the needs of the district, provided a community education experience for PSHMC nurse leaders, and offered an opportunity to address overweight and obesity among Pennsylvania children, while supporting the needs assessment priority area “promotion of healthy lifestyles.”

Planning for the screening events took 1 year (Table) and included several regular meetings between district administrators, school nurses, and project personnel from PSHMC. The following factors were considered: a timeline for training nurses in how to conduct the screenings, availability of screening equipment, and capacity of PSHMC nursing staff. The planning team selected dates for a 2-day screening of students in all 7 district schools. Each school identified a screening location, and the district’s technology department established a plan to provide computers and printers in the screening location for entering data into a database and printing parent notification letters.

**Table:** Process of Planning and Implementing Lebanon Student Health Screenings, Lebanon, Pennsylvania, 2012–2013

Activity	2012							2013	
	Jun	Jul	Aug	Sep	Oct	Nov	Dec	Jan	Feb
Lebanon School District is identified as partner	x								
PSHMC support for project is obtained		x							
Lebanon School District support for project is obtained		x							
Planning meetings with Lebanon School District and PSHMC take place		x	x	x					
PSHMC experts provide consultation for methodologically sound screenings		x	x						
PSHMC nurses are trained in screening methods			x						
Schedules for staff and schools are developed				x	x				
Screenings take place							x		
Debrief meetings and follow-up surveys are completed								x	x

Abbreviation: PSHMC, Penn State Hershey Medical Center.

PSHMC experts in nursing, ophthalmology, audiology, and weight management collaborated with project staff to ensure standard measurement methods and technologies were incorporated into screening plans. A PSHMC nursing team was organized to perform the screenings. Each nurse viewed a standardized 1-hour video, which included a review of anatomy and physiology, and participated in hands-on skills training for each type of screening they would be performing. Training was developed according to Pennsylvania Department of Health screening guidelines (12–15). All hospital personnel who planned to be on-site for the screenings signed a confidentiality form and showed proof of their state-required criminal background checks.

In December 2012, health screenings for height, weight, scoliosis, vision, and hearing were conducted according to regulations of the Pennsylvania Department of Education (11). PSHMC nurses and school nurses used standardized screening protocols and equipment. Each school’s screenings took place during 1 school day; students visited a separate station for each screening, beginning with height and weight measurements (because those are the fastest), and they rotated through stations as stations became available. All screening stations, except for hearing, were set up in the school’s gymnasium or auditorium. Hearing screenings were conducted in the school’s library to reduce competing noises. To complete all screenings at 1 school in 1 day, 1 nurse was needed for every 50 students.

Elementary and middle school students reported to the screening areas by grade and classroom. For logistical purposes, high school students reported to the screening area alphabetically by last name. Students were given a personalized paper form to carry with them to each station. To ensure student privacy, nurses recorded the screening results on the forms, and all forms were collected before the student exited the screening area. In addition, adequate space was maintained between the student being screened and those waiting in line to be screened; privacy screens were not required or used.

PRO Wellness staff members electronically entered data on height and weight during each screening event, and BMI was calculated automatically in Health eTools software (16). Each student’s data were entered using a double data entry check method. Letters notifying parents of their child’s BMI were generated through Health eTools (16). Parent letters were printed and placed into pre-addressed envelopes. For vision, hearing, and scoliosis screenings, only parents of students who required a referral were notified by letter, whereas all parents were notified of their child’s height, weight, and BMI. To ensure student privacy and compliance with the Family Educational Rights and Privacy Act, the name of the student in the letter was matched to the information printed on the envelope, and all letters were mailed from the school at the completion of each school’s screening event (17).

## Outcomes

Annual health screenings were completed for 3,105 students (67% of all students) from all 7 schools. Parents of each student received screening results early enough in the school year to allow them to take any necessary action. Additionally, school nurses were relieved of a task that typically spanned the length of a school year, creating an opportunity for them to focus on other areas of school health.

Several strategies were used to debrief partners and measure success. School nurses participated in a meeting to discuss the screenings and identified the need for training or guidance materials to assist them in assessing and leading overall wellness initiatives in their school. In addition, the PSHMC nursing team and school nurses were invited to participate in a questionnaire on items such as training, screening methods, adequacy of the screening equipment, and their ability to answer students’ questions. A final meeting with district administrators, school nurses, and key project personnel from PSHMC and PRO Wellness was arranged to plan improvements for subsequent screening years.

One unexpected outcome of the screening events was the discovery that 41.5% of students were overweight or obese, compared with 38% in previous years (10). Student BMI may have increased, but another possibility is that using standardized screening protocols resulted in more accurate data on height and weight. Additional research is needed to fully explore this possibility.

Several aspects of preparation aided in the success of the screening events and subsequent dissemination of notification letters to parents. These aspects were 1) preprinting and presorting mailing envelopes by classroom, 2) using multiple stations for each type of screening to improve student flow, and 3) having adequate numbers of nurses and staff members available at the screening events. Areas requiring particular attention are 1) assurance that screening equipment is up to date, especially equipment used for screenings that require more time than other screenings (eg, vision), 2) verification that equipment is in working order, 3) calibration of hearing equipment, and 4) use of a stand-alone stadiometer (instead of stadiometer–scale) for measuring height.

Each student age group had its unique challenges. High school students were asked via school intercom to report to the screening area and were unaccompanied by a teacher. This method reduced the number of students who actually reported for their screenings, thereby reducing overall student participation. In addition, high school students were released to the screening location by last name instead of by grade and classroom. Because the software used to enter the screening data and generate parent notification letters organized students by classroom, not by student last name, having high school students arrive alphabetically increased the time required to locate the student in the system, enter the data, generate the letter, and ensure a proper match between the letter and the pre-addressed mailing envelope. Middle school students compared their screening results with their peers, especially their BMI results, creating a potential opportunity for ridicule or bullying. The largest challenge among young elementary school students was their inability to remain quiet during hearing tests. In addition, a language barrier created difficulties in screening students who did not speak fluent English. Many students failed to bring their glasses to the vision test, a common challenge among all age groups.

## Interpretation

Overall, the collaboration with the local school district was successful in completing most required student health screenings in 2 days. Absenteeism, failure to report to the screening area, not having glasses, and language barriers prevented 100% participation and required school nurses to complete missed screenings at another time. With a large portion of the screenings completed early in the school year, more time was made available for school nurses to address daily acute student health concerns and troubleshoot student health concerns identified by the screenings. Because school nurses spent less time on screening, they had more time to take on the role of school wellness champion. In addition, parents found out earlier in the school year whether their child needed physician follow-up for vision, hearing, scoliosis, or weight-related issues.

Collaboration created the opportunity for addressing health needs in an expanded capacity. It cultivated a trusting relationship between PSHMC and the Lebanon community, thus overcoming a barrier often present between academic medical centers and their surrounding communities (18). Through this collaboration and the discovery of a higher-than-expected rate of overweight and obesity, PSHMC, PRO Wellness, and district administrators planned to extend outreach activities to include school health environment assessments and education on healthy lifestyle choices in addition to annual school screening events. The collaboration also opened up a research opportunity to improve screening methods for hearing. Other collaborative activities now underway are evaluation of the BMI parent notification letter, nutrition and physical activity interventions for students, and school nurse educational conferences.

Although the screening project was largely successful, several items should be considered for future events. Since the inaugural screening event, many of the following suggestions were incorporated as standard practice by the collaboration. First, we suggest that screenings be completed near the beginning of the school year, when absenteeism rates are lower than other times of the year. Screenings scheduled near the December holidays may have contributed to absenteeism. We recommend that data be entered into the database by screening facilitators after, not during, the screening day. Separating the screening event from data entry could increase the number of people available to conduct the screenings and allow for a more streamlined data entry process. This process should include a quality review of data to identify outliers before entry into the database. Special consideration should also be given to how data are recorded during the screening event; the metrics used to record screening data should be consistent with the database used to house the data and the software used to generate parent notification letters (eg, height data recorded as feet and inches or total inches). The order in which the screenings are completed should ensure student privacy. Conducting height and weight screenings last and collecting the completed data forms at that time may decrease the opportunity for students to compare sensitive health information with their peers. We also recommend measuring height and weight behind privacy screens.

The screening events also offer an opportunity for health education. While students wait in screening lines, they can be given age-appropriate health education lessons to reinforce the importance of the screenings. Informing students about the purpose of screening may also reduce the possibility of any stigma or bullying related to screening results. In addition, school nurses should be provided resources to champion wellness initiatives, such as tools for assessing their school environment, developing a wellness council, or reviewing and revising wellness policy.

We have described how collaboration between a nonprofit hospital and local school district facilitated the provision of a state-mandated requirement in a timely and efficient manner. The collaborative screening event contributed to accomplishing the community health needs assessment’s priority of “promotion of a healthy lifestyle,” thereby creating a win–win situation for both the school district and medical center. In addition, the higher-than-expected rates of overweight and obesity found by the screening process opened doors for additional community-engaged research on this public health concern. Partnerships between school districts and nonprofit hospitals to conduct required student health screenings are a practical option for increasing community outreach while satisfying critical community needs.

## Figures and Tables

**Table:** 

**1. The activity supported the learning objectives.**				
**Strongly Disagree**				**Strongly Agree**
1	2	3	4	5
**2. The material was organized clearly for learning to occur.**				
**Strongly Disagree**				**Strongly Agree**
1	2	3	4	5
**3. The content learned from this activity will impact my practice.**				
**Strongly Disagree**				**Strongly Agree**
1	2	3	4	5
**4. The activity was presented objectively and free of commercial bias.**				
**Strongly Disagree**				**Strongly Agree**
1	2	3	4	5

## Post-Test Information

To obtain credit, you should first read the journal article. After reading the article, you should be able to answer the following, related, multiple-choice questions. To complete the questions (with a minimum 75% passing score) and earn continuing medical education (CME) credit, please go to http://www.medscape.org/journal/pcd. Credit cannot be obtained for tests completed on paper, although you may use the worksheet below to keep a record of your answers. You must be a registered user on Medscape.org. If you are not registered on Medscape.org, please click on the "Register" link on the right hand side of the website to register. Only one answer is correct for each question. Once you successfully answer all post-test questions you will be able to view and/or print your certificate. For questions regarding the content of this activity, contact the accredited provider, CME@medscape.net. For technical assistance, contact CME@webmd.net. American Medical Association's Physician's Recognition Award (AMA PRA) credits are accepted in the US as evidence of participation in CME activities. For further information on this award, please refer to http://www.ama-assn.org/ama/pub/about-ama/awards/ama-physicians-recognition-award.page. The AMA has determined that physicians not licensed in the US who participate in this CME activity are eligible for ***AMA PRA Category 1 Credits*** ™. Through agreements that the AMA has made with agencies in some countries, AMA PRA credit may be acceptable as evidence of participation in CME activities. If you are not licensed in the US, please complete the questions online, print the AMA PRA CME credit certificate and present it to your national medical association for review.

## Post-Test Questions

### Article Title: Improving Community Health While Satisfying a Critical Community Need: A Case Study for Not-for-Profit Hospitals

**CME Questions**

1. Your practice is considering participation in a health screening program at a local school district, with screening events for children and adolescents in elementary school through high school. Which of the following screening examinations is required by most school districts in the United States?
  - Body mass index
  - Waist circumference
  - Hearing and vision
  - Scoliosis
2. Which of the following statements regarding the procedures of health screening in the current study is *most* accurate?
  - Most of the screening was performed by medical students
  - Screening was performed for 1 week at each school site
  - The ratio of students to screening health professional was approximately 50:1
  - Screening was performed in classrooms to improve efficiency
3. What percentage of the overall student body of the Lebanon School District was screened as part of the current intervention?
  - 12%
  - 67%
  - 88%
  - 99%
4. Which of the following were recommendations for improving the screening program described in the current study?
  - Screening should be performed at the end of the academic year
  - Data entry should be performed simultaneously with screening to ensure the accuracy of what is recorded
  - Height and weight screenings should be completed last
  - Height and weight screenings should be performed in group settings to increase efficiency

**Evaluation**
